# Development and validation of a cost-effective DIY simulation model for McDonald cerclage training

**DOI:** 10.1007/s00404-024-07812-8

**Published:** 2024-11-14

**Authors:** Johanna Buechel, Adam Kalisz, Saskia-Laureen Herbert, Anne Scherer-Quenzer, Bettina Blau-Schneider, Teresa Starrach, Katrina Kraft, Achim Wöckel, Ulrich Pecks, Matthias Kiesel

**Affiliations:** 1 Department of Obstetrics and Gynecology, University Hospital Würzburg, Josef-Schneider-Str. 4, 97080 Würzburg, Germany; 2 Maternal Health and Midwifery, Julius-Maximilians-University, Würzburg, Germany; 3 Department of Electrical, Electronic and Communication Engineering, Information Technology (LIKE), Friedrich-Alexander-Universität Erlangen-Nürnberg, Erlangen, Germany; 4 Department of Obstetrics and Gynecology, University Hospital Würzburg, Würzburg, Germany; 5 Department of Obstetrics and Gynecology, University Hospital, LMU Munich, Munich, Germany; 6 University Clinic of Gynecology and Obstetrics, Hospital St. Hedwig of the Order of St. John, Regensburg, Germany

**Keywords:** Surgical training, Cervical stitch, Preterm birth, Skills training, High-risk pregnancy

## Abstract

**Purpose:**

The prevention of preterm birth is a challenging task for obstetricians. Cervical cerclage, used as both a primary and secondary prevention method for spontaneous preterm birth, is a crucial surgical intervention. It is essential that obstetricians can learn this procedure in a simulated environment before performing the stitches on high-risk patients. This study aimed to develop a simulator based on 3D printing and evaluate its validity for clinical training.

**Methods:**

The objectives of this study were (1) to design and construct a cost-effective simulator for McDonald cerclage with two different cervix models—a closed cervix and a cervix with bulging membranes—using common material from a DIY store and 3D printing technology and (2) to validate its effectiveness through feedback from learners and experts in cervical cerclage. The self-made simulator was evaluated by obstetricians using a questionnaire with Likert scale.

**Results:**

Obstetricians and gynecologists assessed the simulator and found it useful for learning and practicing cervical cerclage. The simulator was deemed valuable for skill training.

**Conclusion:**

Cervical cerclage is a complex procedure that should be mastered through simulation rather than initial practice on real patients. Our simulator is a cost-effective model suitable for various clinical settings. It has been validated by obstetricians for both preventive and therapeutic cerclage, demonstrating its efficacy for training in cerclage techniques. Future research should focus on less skilled obstetricians and gynecologists and investigate how repeated use of the simulator can enhance their performance in cerclage stitching.

## What does this study add to the clinical work

**Table:** 

The McDonald cerclage simulator is an excellent way to practice McDonald cerclage stitching in a safe setting. Our model provides a cost-effective option that still closely approximates real-life conditions.	

## Introduction

Preterm birth is a global health problem affecting 6–12% of all babies and preventing it remains a significant challenge in perinatal medicine [1]. Premature birth contributes significantly to perinatal morbidity and mortality. Within various causes, spontaneous preterm birth accounts for 70% of preterm deliveries [2]. Cervical cerclage is an established technique for preventing preterm birth: a Cochrane review with 15 studies showed a reduction of premature birth but a non-significant reduction of perinatal mortality [3]. It can be used prophylactically for pregnant women with a history of preterm birth or mid-trimester miscarriage, for those with a shortened cervix as detected by sonography, or as a “rescue method” and secondary prevention when the cervix is already open with bulging membranes [4–6]. The most used techniques are the Shirodkar and the McDonald cerclage with no clear advantage for one technique [7, 8]. The McDonald technique is a purse-string suture around the cervix whereas the Shirodkar technique involves colpotomy and bladder dissection to gain a higher suture placement [9].

The surgical procedure for inserting a cerclage needs training and training occasions are sometimes difficult, as suitable patients often come to the hospital unpredictably and the procedure is not performed electively. Even in hospitals with a large number of high-risk pregnancies, the rate of patients requiring a cervical cerclage is low, so the procedure is rarely performed. Moreover, there is a considerable risk of complications such as premature rupture of membranes, bleeding, infections, and induction of labor which can subsequently lead to pregnancy loss. As the procedure affects both the mother and the unborn child, it can be considered a very stressful situation, and therefore, belongs in experienced hands.

Therefore, the simulation models for cerclage placement are highly valuable for perinatal units to train their staff. In surgical specialties, simulation is widely used for various procedures, such as laparoscopic training [10]. In obstetrics, there are examples like simulation models for training in cesarean section with impacted fetal head [11], vaginal-operative birth [12] or shoulder dystocia [13]. Vaginal surgery, with its challenging access, seems ideal for simulation training [14].

In this study, we describe the design process and construction of a McDonald cerclage trainer based on 3D-printed cervical models and evaluate its suitability for training purposes as assessed by skilled experts and advanced learners in the field.

## Materials and methods

This study includes the development of the simulator and its validation by cerclage experts and trainees. A certificate of non-objection was obtained from the Ethics Committee of the University Hospital Wuerzburg (application number 2024030502).

### Simulator design and development

The Wuerzburg Cervical Cerclage Simulator (WCCS) was developed by two obstetricians and a computer scientist using common materials from a Do it yourself (DIY) store and cervix models. The production of the latter based on 3D printing and molding silicone.

A drainpipe (Marley Deutschland GmbH, Wunstorf, Germany) with a 10 cm diameter was used to simulate the vagina, with foamed plastic (isopur, softpur GmbH, Goellheim, Germany) mimicking the vaginal walls. The drainpipe was mounted on a base plate with the size of 40 × 30 cm (Wibo Kunststofftechnik GmbH, Meitingen) utilizing screws and super glue. The base plate could be angled relative to a second base plate to which it was combined with screws. Suction cups were used to secure the model on a desktop, and two bar clamps could be used additionally to fix the simulator on a desk or an examination couch.

**Fig. 1:**
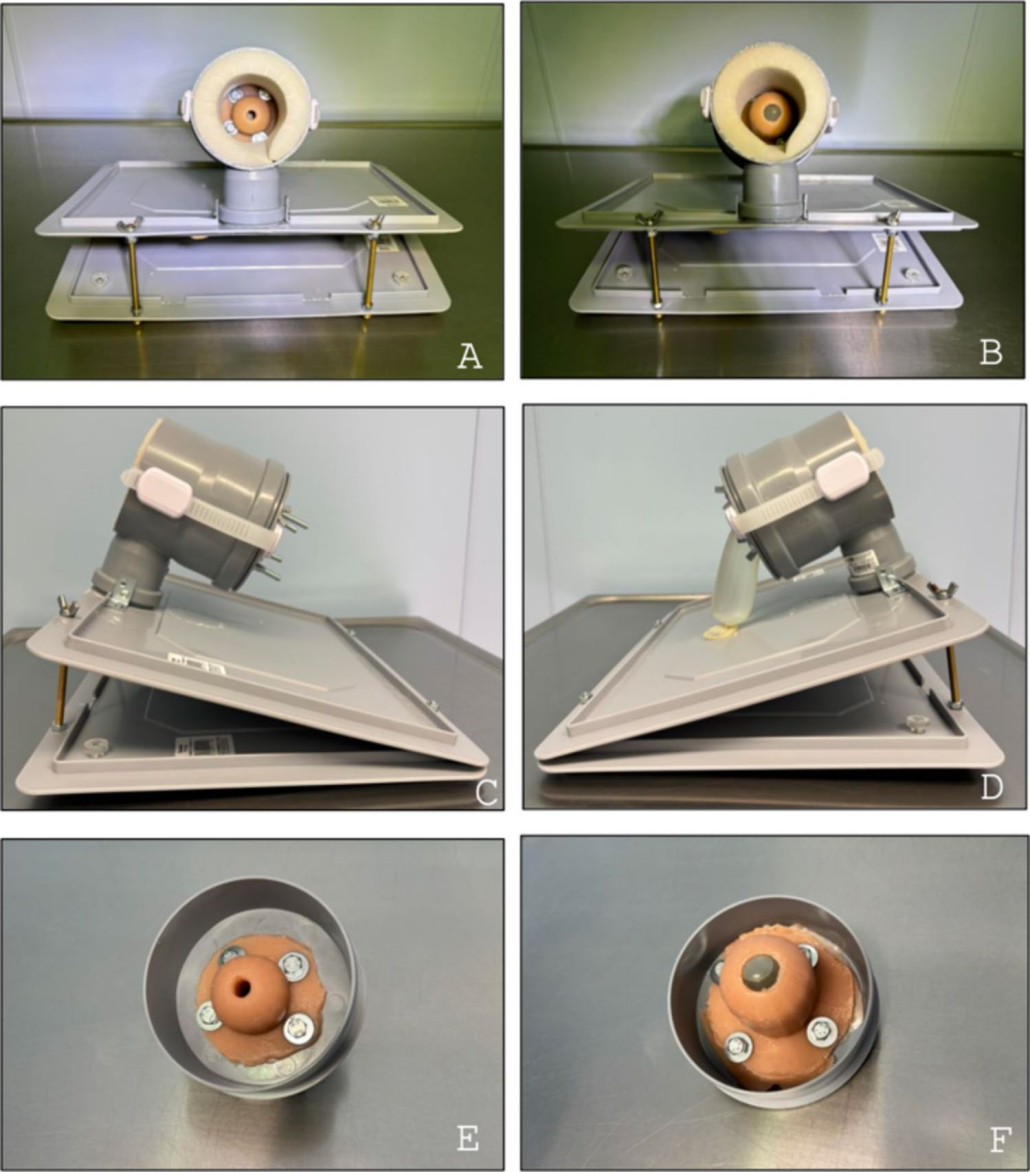
Wuerzburg cervical cerclage simulator (WCCS) presented from different perspectives, with closed cervix (**A**, **C**, **E**) and with open cervix with bulging membranes (**B**, **D**, **F**)

The cervices were first created as virtual models using an open-source 3D modeling program (Blender, version 4.2). Individual parts were then 3D printed using Selective Laser Sintering (SLS) with the Formlabs Fuse 1 (Formlabs, Somerville, USA, Formlabs with thermoplastic polyurethane (TPU) (Formlabs, Somerville, USA). Silicone was used to duplicate the printed cervix models to replicate realistic tissue. In order to create the negative form, the 3D printed cervices were coated with silicone mold separating cream (Troll Factory, Riede, Germany) and then covered with modeling silicone (Laurenz + Morgan GmbH, Bad Salzuflen, Germany). After hardening, the 3D printed cervices were removed. The negative footprint was again coated with silicon mold separating cream and filled with clear silicone (Troll Factory, Riede, Germany). Two different cervix models were printed. The first model was a closed cervix with a length of 5 cm, intended for performing prophylactic cerclage. The second model represented a cervix already opened to 1.5 cm with bulging membranes. The bulging membranes were simulated using a water-filled balloon. Both cervices were duplicated using silicone. For the closed cervix, silicone with a shore hardness of 00 was used. The cervix with bulging membranes was created with silicone with a shore hardness of 10 [15]. For a more natural appearance, the cervices were painted pink according to Deutsches Institut für Gütesicherung und Kennzeichnung e.V. (RAL) (RAL 3012, Troll Factory, Riede, Germany). Steps of the production process as well as the finished simulator can be seen in Fig. 1.

The production cost of the simulator was calculated to be 39.64 EUR, as depicted in Table 1.

**Table 1:** Production costs for one simulator with one cervix

Materials	Amount	Cost per piece (EUR)	Cost total (EUR)
Drain pipe	1	4.39	4.39
Base plate	2	4.99	9.98
Foam f	1	0.94	0.94
Super glue (half tube)	1	3.75	3.75
Plain washer	8	0.06	0.48
Nut	4	0.14	0.56
Wing nut	8	0.65	3.90
Screws	10	0.69	6.90
Mounting of cervix	1	1.25	1.25
Attachment for mounting of cervix	2	1.25	2.50
Suction cups	4	0.7	2.80
		Sum	37.45

The running costs include the material costs for the silicone cervices as well as the suture material. The silicone and paint for the closed cervix costs approximately 0.85 EUR per piece. The running costs for the opened cervix are approximately 2.28 EUR per piece with each cervix usable for approximately five to 10 cerclage procedures. 0.02 EUR are required for the water-filled balloon reassembling the bulging membranes. The time to build one simulator as showed in Fig. 1 was 51 min and 34 s. This was documented with a stopwatch. The experts used their common reusable instruments and performed the McDonald cerclage in the manner they had previously learned.

### Validation

Obstetricians from four different university hospitals in Germany were asked to test the cerclage simulator. They completed a questionnaire that included demographic information, their experience in assisting and performing McDonald cerclage, and feedback on the model’s realism and surface feel. Satisfaction with the removal of the cerclage was also assessed. The questionnaire included questions on a 7-point Likert scale, along with space for free-text comments. Expert opinions were sought. Microsoft Excel (Microsoft, Redmond, USA) was used for descriptive statistics to analyze the questionnaire results. Figure 4 was created using “R” (r-project.org).

## Results

### Simulator use

**Fig. 2:**
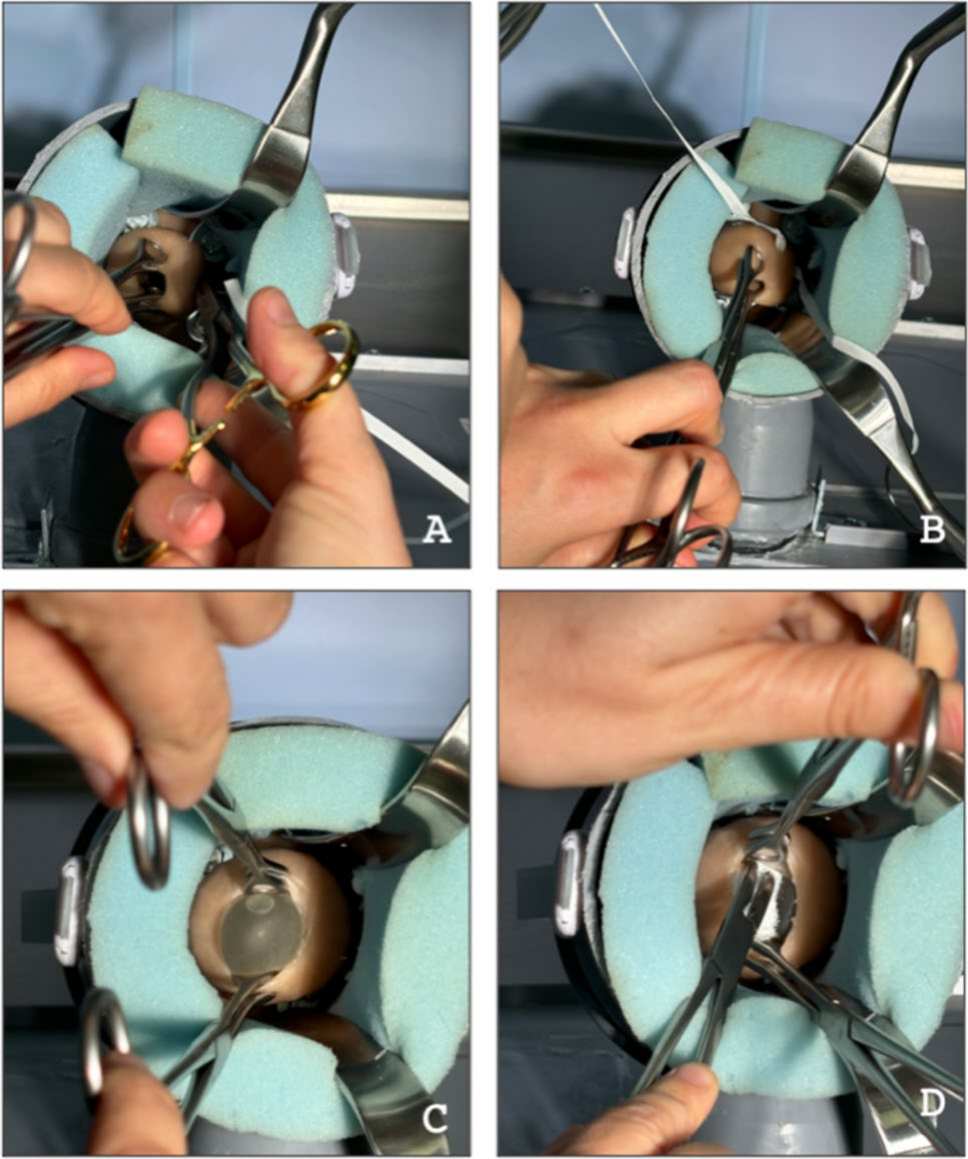
Procedure of McDonald cerclage performed with standard instruments on the WCCS which realistically simulates the spatial conditions of a vaginal surgery. **A**, **B** Stitching with the help of a Breisky vaginal retractor. **C** Cervical hooking of the open cervix with bulging membranes. **D** Use of a sponge forceps with gauze to push back the amniotic sac

Transporting the simulator to the various training locations was easy, even by public transport, as it is a compact and lightweight design with a total weight of 1110 g. The simulator was used in various environments, including an examination chair, a delivery table and a writing desk. A headlamp or the examination light in the delivery room served as the light source. The procedure was performed with standard instruments used across various hospitals, including a Breisky vaginal retractor, needle holder, sponge clamps, Korn forceps, swabs, and a 5-mm braided suture (Ethicon Mersilene BP-2 5 mm 40 cm). The WCCS during use is shown in Fig. 2. Cervix models were replaced after multiple uses and to test both the closed cervix and the open cervix with bulging membranes.

### Feedback

**Fig. 3:**
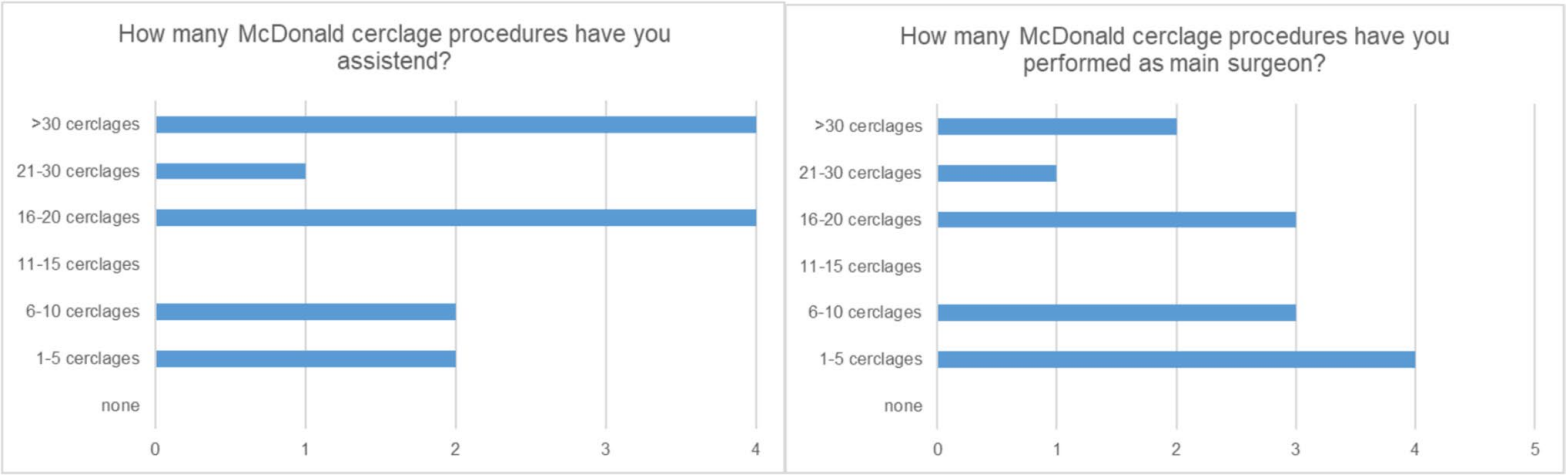
Level of experience among the 13 obstetricians who participated in the study. Left: amount of McDonald cerclage procedures assisted. Right: McDonald cerclage procedures performed

Thirteen obstetricians with experience in performing McDonald cerclage evaluated the simulator. Among them, three experts serve as department heads, six as senior physicians, three as consultants, and one as a resident. Each participant had prior experience assisting and performing McDonald cerclage in clinical settings, varying in frequency based on their respective levels of experience (see Fig. 2). Six participants had performed more than ten McDonald procedures by themselves and can be called “experts”, whereas seven hat performed less than ten (“trainee status”) (Fig. 3).

**Fig. 4:**
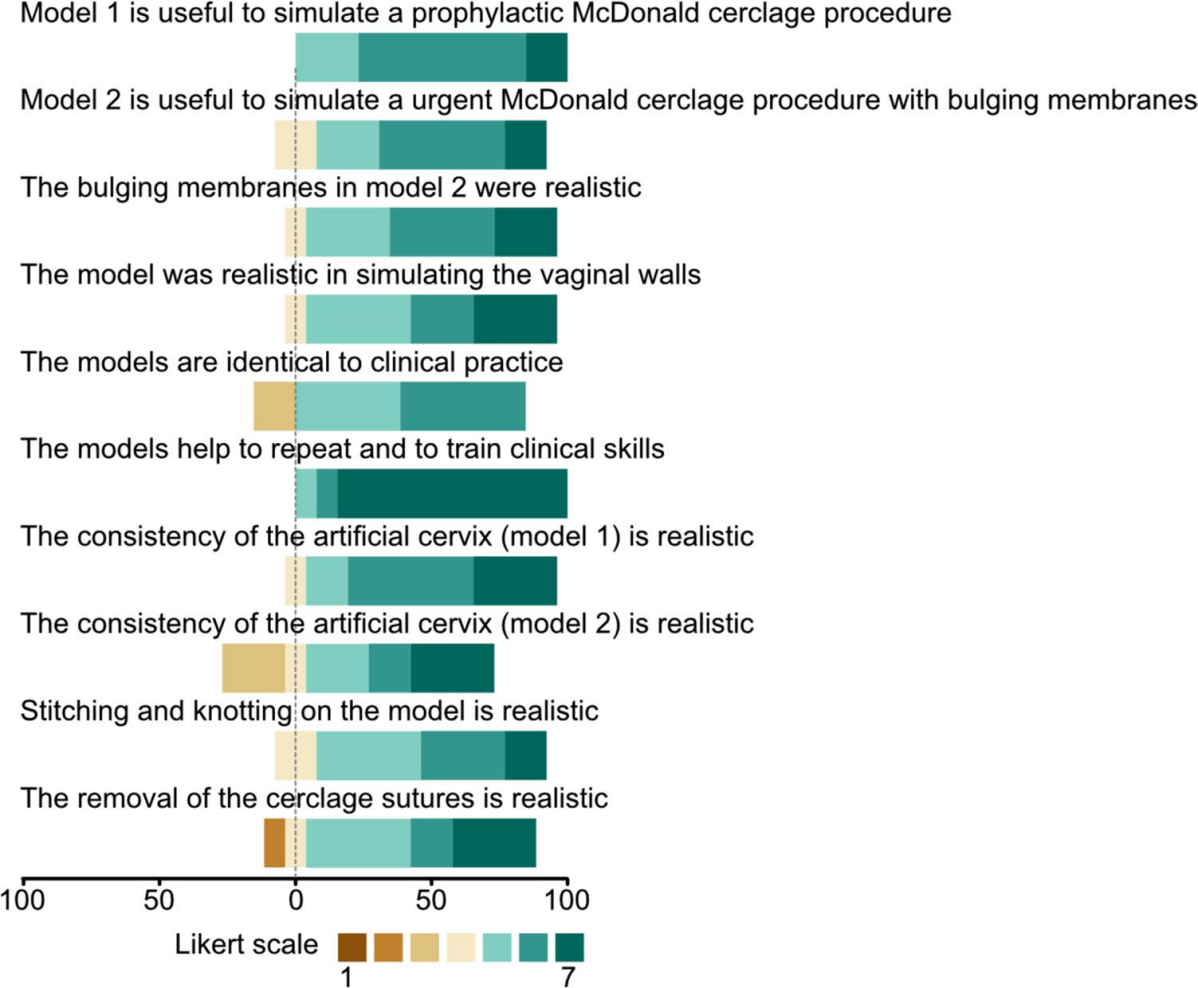
Participants were asked for their feedback concerning different aspects of the simulator using a 7-point-Likert scale where 1 is “very strongly disagree” with color code in brown and 7 is “very strongly agree” in color code of dark green. The length of each color segment within a bar shows the percentage of respondents who gave that specific rating. The zero line indicates neutrality towards the statement

All participants were asked to evaluate the simulator using both a closed cervix and subsequently an open cervix with bulging membranes. Figure 4 summarizes the feedback on various aspects using a 7-point Likert scale. The simulation of a prophylactic McDonald cerclage was rated Mean 5.92 (standard deviation 0.67), and simulation of an open cervix was rated Mean 5.75 (standard deviation 0.87), bulging membranes Mean 5.83 (standard deviation 0.94).

All participants noted that the simulator was effective for training stitches for McDonald cerclage (rating Mean 6.92, standard deviation 0.29), providing a high degree of realistic challenges, despite the simulator material not meeting all criteria of real tissue. Positive free-text comments facilitated minor ideas for improvement of the simulator.

We asked the participants, if they enjoyed the training. On a 7-point Likert scale, the Mean was 6.67 (standard deviation 0.89). Additionally, all participants expressed high interest (Mean 7.00) in using the cerclage simulator in their unit to train both novices and experts in McDonald cerclage techniques.

## Discussion

Reducing preterm delivery is a common goal in perinatal medicine and interventions such as cervical cerclage, often combined with progesterone, appear to aid in prolonging pregnancy [3, 16]. Shirodkar cerclage was first reported in 1955 [17] and underwent some modifications. It includes colpotomy and bladder dissection. McDonald published his technique in 1957 [18]. This technique avoids a dissection of bladder and rectum and became, nowadays, the most performed cerclage technique. Until 2024, there is no well-designed study confirming a superiority of either technique. As the McDonald cerclage is considered to be easier in placing the stitches and removing the material for birth, most favor this technique [19]. In 2023, a systematic review and meta-analysis stated that pregnancies with a Shirodkar cerclage were less likely to result in preterm birth in comparison to McDonald cerclage but with a significant limitation of bias of many of the included studies [7]. The choice of cerclage technique remains to be at the discretion of the surgeon [8]. The effectivity of McDonald cerclage seems to be dependent on the height of the stitches with at least 18 mm from the cerclage to the external OS which is not always feasible [20–22]. As the placement of McDonald cerclage is, even if it is less demanding than the Shirodkar procedure, a technically demanding skill and the height of the knot is of importance, the WCCS can be a training option to increase the height of the knot. The need for simulation is clear as it should not be initially learned on patients. The use of simulation training in obstetrics is already investigated in different procedures [11, 13, 23, 24]. Simulation training for rare, high-risk surgeries increases patient safety and boosts the confidence of obstetricians. Cervical cerclage simulators address this specific need. Skills can be acquired using various simulation models, as this procedure is challenging to teach and learn. Therefore, we developed a cost-effective DIY simulator, the WCCS, using 3D printed cervix models to train for different clinical scenarios. Evaluation by obstetric experts and trainees was promising.

Overall, the experts surveyed were satisfied with the use of the model. They reported that they enjoyed the training and all expressed their willingness to use the simulator in training sessions for their teams. The learners with an experience of less than 10 McDonald cerclages done by themselves, reported even higher rates of satisfaction with the session.

The literature reports various other approaches to creating a cerclage simulator. Hall et al. developed a model used in a commercial training phantom from Limbs & Things [25]. This model relies on a commercial pelvic trainer, which is expensive and makes learners dependent on a commercial system. Hospitals rarely provide multiple pelvic simulators to facilitate simultaneous training for several participants. A commercial cervix system can be used approximately 18 times with estimated 0.90 £ (1.07 EUR) required per training [25]. In contrast, our model can be used about five to 10 times with costs per training varying between 0.09 and 0.46 EUR. Hence, less acquisition and running costs are generated.

Other studies refer to models made from plastic bottles and additional material like hair buns [26, 27], or models using polyvinyl chloride piper and frozen cow muscle [28]. While some of the materials are inexpensive, obtaining frozen bovine tissue can be challenging, and using biological material has limitations and cannot be stored for several teaching sessions.

A limitation of the developed cervical cerclage simulator is that the materials used do not accurately replicate the haptic properties of cervical and vaginal tissue and could negatively impact the learners' performance. The decision on which silicone shore hardness to use for each model was made by balancing the tactile feel with the interaction between the cervix model and the seam material. The shore hardness of 00 used for the closed cervix comes closer to real-life conditions and was, therefore, rated better by the participants of the study. For the open cervix with bulging membranes, the used silicone shore hardness of 10 allowed not only for putting the stitches but also for tightening and tying the suture whereas the smoother material probes (shore 00) for the open cervix were not useful as a tearing of the sutures was common.

However, our expert panel at least considered it realistic enough to train the trainees. Trainees with experience in less than 10 cerclage procedures valuated the simulator even better regarding the tissue than experts. Perhaps, they focus more on the basic technique in cerclage placement and are focusing on putting the stitches in the right manner rather than in tissue differences as they have less experience with complicated cases with weak tissue.

Another limitation is that the WCCS is only suitable for the McDonald’s technique, while there is still no consensus, but some studies favor the Shirodkar cerclage [7]. As the McDonalds cerclage is easier to learn, this technique is the first choice for trainees.

There has been a discussion about the suture material for McDonald cerclage [29, 30]. A randomized controlled trial in the UK did not find a difference between braided and monofilament suture. We tested our model only with Mersilene braided sutures. As the silicone shore hardness for our models reacted very different in a test phase, we decided to optimize the model for the braided suture because it is used more frequently [31].

A consensus about the best modified method to perform a McDonald cerclage is still missing although the initial procedure was established in 1957 [18]. We aimed to get a broad feed-back from obstetricians in different hospitals and settings to test our simulator. Further studies could focus on establishing a standard procedure with our McDonald cerclage simulator considering actual findings like knot placing or height of the cerclage [32]. The WCCS can be used for identifying differences regarding surgical technique.

Our presented simulator should next be tested with novices. In this context, the markers developed by Hall et al. [25] could be evaluated using our model to demonstrate a learning curve concerning metrics as stitch position, cerclage height, cerclage circumference, or destruction of the bulging amniotic sac. Afterwards, translation to the clinical setting can be studied.

## Conclusions

McDonald cervical cerclage is a crucial yet complex procedure in obstetrics aimed at preventing preterm birth. Given the limited training opportunities and the high-risk nature of the procedure, simulation emerges as a valuable tool for training obstetricians. We introduce a resource-effective DIY simulator that can be constructed using commercial and 3D printing materials to simulate different cervix scenarios. Further research, particularly focusing on novice learners, is essential to validate the efficacy of regular simulator use in enhancing technique and improving patient outcomes.

## Data Availability

Raw data are available on request.
